# An Integrated Transient Expression and UPLC‐PDA Approach for Functional Genomics and Metabolic Analysis of *Cannabis sativa*

**DOI:** 10.1002/biot.70316

**Published:** 2026-10-14

**Authors:** Sarah Pullano, Aida Shahabi, Parmis Shariat Torbaghani, Ivan Monsalvo, Madison Sandler, Adam Sumner, Alex Harris, David Haak, Bastiaan Bargmann, Nik Kovinich

**Affiliations:** ^1^ Department of Biology York University Toronto Ontario Canada; ^2^ College of Agricultural and Life Sciences Virginia Tech Blacksburg Virginia USA

**Keywords:** *Agrobacterium tumefaciens*, *Cannabis sativa*, RUBY reporter system, transient expression, trichomes

## Abstract

*Cannabis sativa* (*Cannabis*) is a high‐value medicinal crop whose functional genomics and metabolic engineering remain constrained by limited genetic tools. The species is regarded as recalcitrant to stable transformation, and existing transient expression systems exhibit low efficiency or are restricted to hemp cultivars. Here, we report two *Agrobacterium*‐mediated agroinfiltration protocols enabling rapid, robust, and reproducible transient gene expression in drug‐type, high‐THC *Cannabis* tissues. Using the *RUBY* betalain reporter system, we demonstrate transformation efficiencies of up to 100% in mature leaf disks and 81%–100% in whole seedlings. By independently comparing *Agrobacterium* strains and vector configurations, we demonstrate that transformation efficiency and transgene expression are influenced by distinct experimental variables, with EHA105 providing the highest transformation efficiency and *p35S*‐based vectors producing the strongest reporter expression. Complementing these genetic tools, we developed a UPLC‐PDA method enabling simultaneous detection and quantification of cannabinoids and cannflavins. While not intended to replace mass spectrometry‐based analyses, the method provides an accessible and cost‐effective platform for comparative metabolite profiling during functional genomics and metabolic engineering studies. Together, these advances establish an integrated workflow linking high‐efficiency transient gene expression with streamlined biochemical analysis, providing a practical foundation for gene function studies, pathway interrogation, and biotechnology‐driven trait engineering in *Cannabis*.

## Graphical Abstract and Lay Summary

*Cannabis* research is limited by a lack of efficient tools for studying gene function and metabolism. We developed two rapid, high‐efficiency methods for transient gene expression in high‐THC *Cannabis* and a simple, cost‐effective method to measure cannabinoids and cannflavins. Together, these complementary tools provide an accessible platform for investigating gene function, specialized metabolism, and future crop improvement. Graphical abstract created using BioRender.

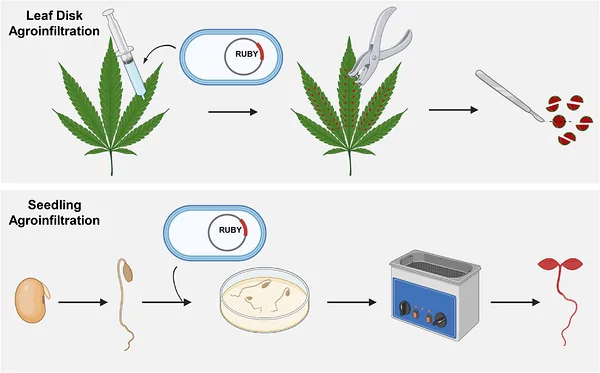

## 1 Introduction

*Cannabis sativa* (*Cannabis*) is a high‐value medicinal crop distinguished by its capacity to synthesize high amounts of important specialized metabolites, including cannabinoids, terpenoids and cannflavins. Cannabinoids are produced predominantly in the glandular trichomes of female inflorescences and can accumulate to exceptionally high levels in elite cultivars, with Δ^9^‐tetrahydrocannabinol (THC) reaching up to 20%–35% of dry floral biomass [1, 2]. These compounds underpin both the pharmaceutical relevance of *Cannabis* and the growing demand for genetic tools to understand *Cannabis* metabolism [3, 4, 5].

Despite this importance, functional genomics in *Cannabis* remains severely constrained by the absence of reliable genetic transformation methods [6]. The species is widely regarded as recalcitrant to stable transformation [7], limiting the application of conventional reverse‐genetics approaches. Alternative methods, including *Agrobacterium*‐induced hairy root cultures and *Cannabis* protoplast transformation, enable the study of *Cannabis* gene function [8, 9]. Recent advances in plant biotechnology have further expanded the molecular toolbox through improved virus‐mediated gene editing and gene silencing systems, while continued optimization of construct design and transformation strategies has enhanced the efficiency of plant genetic manipulation across diverse species [10, 11, 12]. However, these approaches have limited utility for assessing gene function in specialized metabolic pathways that are restricted to trichomes or other aerial tissues.

Although several stable and transient genetic transformation platforms have been reported for *Cannabis*, they differ substantially in target tissue, approach, delivery system, reporter, cultivar type and efficiency (Table 1). Existing *Agrobacterium*‐mediated transient expression protocols have primarily focused on leaf or seedling tissues of hemp cultivars, and exhibit relatively modest transformation efficiencies, whereas other approaches such as nanoparticle‐mediated delivery require specialized expertise and equipment. Collectively, these limitations have constrained routine functional genomics in commercially relevant drug‐type *Cannabis*.

**TABLE 1:** Comparison of published genetic transformation platforms for *Cannabis sativa*.

Tissue	Approach	Delivery system	Reporter	Cultivar	Efficiency (%)	Study
Suspension cell	Stable	*A. tumefaciens* EHA101	*PMI*	Hemp‐type (UnikoB, Kompolti, Anka, Felina‐34)	1‐2	Feeney and Punja, 2003
Hairy root	Stable	*A. rhizogenes* many strains	*GUS*	Drug‐type (CAN0221)	43‐98	Wahby et al. 2013
Various tissues/ organs	Transient	*A. tumefaciens* GV3101, LBA4404, EHA105	*GUS, PDS*	Hemp‐type (Fedora 17, Felina 32, Ferimon, Futura 75, Santhica 27, USO31, CRS‐1, and CFX‐2)	Not reported	Deguchi et al., 2020
Seedling	Transient	*A. tumefaciens* EHA105	*GUS*	Drug‐type (Nightingale, Holy Grail x CD‐1, and Green Crack CBD)	45‐70.6	Sorokin et al., 2020
Protoplast	Transient	*A. tumefaciens* AGL1	*GFP*	Hemp‐type (YUNMA7)	63	Zhang et al. 2021
Immature embryo hypocotyl	Stable	*A. tumefaciens* AGL1	*PDS*	Hemp‐type (YUNMA7)	2.48	Zhang et al. 2021
Protoplast	Transient	PEG	*GFP*	Drug‐type (high THC)	23.2	Matchett‐Oates et al. 2021
Hypocotyl	Stable	*A. tumefaciens* LBA4404	*GUS*	Hemp‐type (Ferimon, Felina32, Fedora17, Futura75, USO31, FINOLA)	0.8‐5	Galán‐Ávila et al. 2021
Leaf	Transient	Nanoparticle	*GFP*	Hemp‐type (Tygra)	80	Ahmed et al., 2021
Callus	Stable	*A. tumefaciens* EHA105, GV3101	*bar*	Drug‐type (Pink Kush,Copenhagen Kush, Moby Dick)	5‐95	Holmes and Punja, 2023
Leaf	Transient	*A. tumefaciens* GV3101, EHA105, LBA4404, AGL1	*RUBY*, *GFP*, *GUS*	Hemp‐type (Yunma 7) and drug‐type (Galaxy CBD)	0‐60	Li et al., 2024
Hairy root	Stable	*A. rhizogenes* A4, ARqual, K599	*RUBY*	Drug‐type (AJ‐441)	8‐75	Ajdanian and Torkamaneh, 2025
Leaf	Transient	*A. tumefaciens* EHA105 (*pSOUP‐P19*), EHA105 (pSOUP), GV3101, AGL1	*RUBY*	Drug‐type (Megalodon x 50/50 Diesel, Purple Cloud)	52‐100	This Study
Seedling	Transient	*A. tumefaciens* EHA105 (*pSOUP‐P19*), EHA105 (pSOUP), GV3101, AGL1	*RUBY*	Drug‐type (Megalodon x 50/50 Diesel, Purple Cloud)	81‐100	This Study

Here, we report two *Agrobacterium*‐mediated agroinfiltration protocols that enable rapid, robust, and reproducible transient gene expression in trichome‐bearing *Cannabis* tissues. Transformation efficiency was evaluated using the *RUBY* reporter system, a synthetic construct that converts endogenous tyrosine into visible red betalain pigments upon expression [13]. Together, these protocols establish a high‐efficiency, accessible transient expression platform for *Cannabis* functional genomics and biotechnology.

## 2 Materials and Methods

### 2.1 Chemicals

All stock solutions were prepared at 1000 times working concentration and stored at −20°C until use. Stocks of spectinomycin (Gold Biotechnology, St. Louis, MO, USA), kanamycin (PhytoTech Labs, Lenexa, KS, USA), timentin (Caisson Labs, Smithfield, UT, USA), tetracycline and DL‐dithiothreitol (DTT) (Cayman Chemical Company, Ann Arbor, MI, USA) were prepared in MilliQ filtered water at 50, 50, 500, 10, and 150 mg/mL, respectively, and were filter sterilized. Acetosyringone (Cayman Chemical Company) was prepared in dimethyl sulfoxide at 100 mM. Cannflavin A (10 mM) was prepared in acetone. Twenty dilutions (4 nM to 50 µM) of a cannabinoid standards (cannabichromenic acid (CBCA), cannabidivarinic acid (CBDVA), cannabidiolic acid (CBDA), cannabigerolic acid (CBGA), tetrahydrocannabivarinic acid (THCVA), and 2‐carboxy‐tetrahydrocannabinolic acid (THCA‐A); C‐218, Millipore Sigma, Burlington, MA, USA), and cannflavin A (Toronto Research Chemicals, North York, ON, Canada) were prepared in one part 9:1:0.001 methanol:dichloromethane: acetic acid to two parts 3:1:0.001 water:acetonitrile:acetic acid.

### 2.2 *Cannabis* Cultivation

Seeds of the high‐THC strains Megalodon x 50/50 Diesel (MD) and Purple Cloud (PC) were obtained from licensed cultivators. Seeds were sterilized in 15% bleach with continuous rotation for 5 min, then washed with autoclaved MilliQ water in triplicate. Sterilized seeds were germinated in the dark at room temperature for 2 d in liquid DKW medium (Driver and Kuniyuki Walnut nutrients (5.22 g/L; PhytoTech Labs), Murashige and Skoog vitamins (2.5 mL/L; PhytoTech Labs), 30 g/L sucrose, pH 5.7 with KOH) supplemented with 200 µL of 30% (*w*/*w*) hydrogen peroxide.

Germinated seeds were transferred to sterile paper towels saturated with DKW medium and grown in a 16 h light/8 h dark photoperiod at 22°C for 7 d. Seedlings were transferred to soil and maintained in a growth chamber at 20°C and 30% humidity under an 18 h light/6 h dark photoperiod. Plants were watered three times per week for 90 s using an automated watering machine (Deepbang, Dongguan, Guangdong, China). Leaves of 8‐week‐old plants were used for agroinfiltration.

### 2.3 Cloning

The *RUBY* cassette from *p35S::RUBY* (160908, Addgene, Watertown, MA, USA) was amplified by PCR using primers *FattRUBYf‐FattRUBYr* and a high‐fidelity polymerase, then recombined into *pDONR221* using BP Clonase (GeneCopoeia, Rockville, MD, USA). The *RUBY* cassette was subsequently transferred by LR recombination into *pGWB602* downstream of the viral *p35S* constitutive promoter (pdi00088, Riken BRC, Tsukuba, Ibaraki, Japan). The *pGreenII 62‐SK* vector (V012539, Novopro Bioscience Inc., Minhang District, Shanghai, China), a vector whose high copy number is facilitated by *pSoup*, was converted into the Gateway‐compatible *p62‐GW* (henceforth *p35S*) by amplifying the Gateway cassette from *pDONR221* with primers *B2GWf‐B2GWr* and inserting the resultant amplicon into the EcoRV site of *pGreenII 62‐SK*. The *RUBY‐pGWB602* construct was transformed into *Agrobacterium tumefaciens* (*A. tumefaciens*) strains GV3101, AGL1, EHA105 (*pSoup*), and EHA105 (*pSoup‐P19*), while *p35S::RUBY* was transformed into EHA105 (*pSoup*) and EHA105 (*pSoup‐P19*). Transformants were selected on LB agar containing spectinomycin (100 mg/L), or spectinomycin (100 mg/L) plus tetracycline (5 mg/L). Successful transformants carrying the *RUBY* cassette were identified by colony PCR using primers *cRUBY1f‐cRUBY1r* and were fully sequenced to ensure fidelity. All primers used in this research are listed in Table S1.

### 2.4 Leaf Agroinfiltration (the Leaf Disk Method)

Freshly streaked *A. tumefaciens* were incubated for 16 h at 30°C, then resuspended in buffer (10 mM MgCl_2_, 10 mM MES pH = 7.5 by KOH, 100 µM acetosyringone), adjusted to an OD_600_ of 0.8, and incubated in darkness for a minimum of 1 h. Using a 1 mL needleless syringe (Henke Sass Wolf, Dudley, MA, USA), agrobacteria were infiltrated into the abaxial side of a leaf in the interveinal tissues. Four leaves were typically infiltrated per plant. Plants were maintained in darkness overnight, then returned to an 18 h light/6 h dark photoperiod. At 6 d post infiltration, leaf disks were harvested using a ¼ inch hole punch. *RUBY*‐transformed disks were bisected, and 10 half disks were pooled per biological replicate for RNA extraction, with the other half disks being used as a corresponding sample for metabolite extraction. Empty vector control disks were not bisected as no *RUBY* expression was observed in all samples. Disks were flash frozen in liquid nitrogen and freeze dried for 2 d, then either frozen at −80°C, or used directly for RNA or metabolite extraction.

### 2.5 Expression Efficiency Measurements

Images of adaxial sides of leaves were captured using a PowerShot G12 digital camera (Canon, Ota‐ku, Tokyo, Japan). 22–75 infiltrations were performed on each leaf, with three leaves infiltrated per *Agrobacterium* strain. Transformation efficiency was determined by calculating the proportion of infiltration sites exhibiting red pigmentation relative to the total number of infiltration sites. For each biological replicate, all infiltration sites across a single mature leaf (three leaflets) were scored and used to calculate a single transformation efficiency value. Individual infiltration sites were therefore not treated as independent biological replicates. Pigmentation was scored independently by two individuals to assess reproducibility, and red infiltration sites were also counted using ImageJ using an adaptation of the algorithm PIDIQ, with threshold values of 0 and 29 [14]. Transformation efficiency was calculated as: (number of red infiltration sites/total infiltration sites) × 100. An efficiency value was obtained for each leaf, and the mean of three leaves was determined.

### 2.6 Seedling Agroinfiltration

Seeds were sterilized as described above. *Agrobacterium* was resuspended to an OD_600_ of 0.8 in DKW infiltration media supplemented with acetosyringone and DTT. The seed coat and embryo sac were dissected from germinated seedlings. Dissected seedlings were immersed in agrobacteria suspension, sonicated for 30 s, and placed in darkness for 3 h. Upon removal of the agrobacteria suspension, seedlings were placed in empty Petri dishes sealed with parafilm and maintained in darkness for 4 d, with no more than 10 seedlings per dish. A small piece of paper towel saturated with DKW media was placed at the edge of each dish to maintain humidity.

Etiolated seedlings were transferred to liquid DKW media containing half‐strength timentin on a rotary shaker (60 rpm) for 48 h under 22°C and a 16 h light/8 h dark photoperiod, then placed on non‐bleached (brown) paper towels saturated with DKW in a petri dish for an additional 1 to 2 d. Healthy green seedlings were flash frozen in liquid nitrogen, and freeze dried for 2 d, prior to RNA and metabolite extraction.

### 2.7 RNA Extraction and Gene Expression Measurements

Freeze dried tissues (leaf disks or seedlings) were homogenized using a Retsch MM 400 Mixer Mill at a frequency of 30 s^−1^ for 1 min. RNA was extracted using the HiPure Universal RNA Kit (GeneBio Systems, Burlington, ON, Canada), then reverse transcribed to cDNA using a cDNA synthesis kit (GeneBio Systems). qRT‐PCR was conducted on a Bio‐Rad CFX Opus 96 Real‐Time PCR System using GB‐Amp InFluor Green qPCR Master Mix (No ROX) (GeneBio Systems) with cycling parameters of 95°C for 3 min followed by 40 cycles of 95°C for 5 s and 60°C for 30 s. Data was normalized using the equation 2^−[Ct^[15]^−Ct(^ *^CsEF1α^* ^)]^. *CsEF1α* was selected as the reference gene because previous validation studies identified it as one of the most stably expressed reference genes for qRT‐PCR normalization in *Cannabis* across diverse tissues, developmental stages, and stress conditions [16, 17]. In addition, CsEF1α has been successfully used as the reference gene in our previous *Cannabis* gene expression study [18].

### 2.8 Metabolite Extraction and Analysis

For extraction and analysis of betalains, freeze‐dried leaf disks and seedlings were submerged in 40 µL per milligram of dried tissue of extraction solution (80% methanol, 0.1% formic acid), then placed on a rotary shaker at 85 rpm overnight in the dark at room temperature. Extracts were filtered through 0.22 µm PVDF (Millipore Sigma) at 12,000 × g for 1 min. An equal volume of hexanes (Fisher Chemical, Waltham, MA, USA) was added to 50 µL of filtrate, vortexed for 30 s, then briefly microcentrifuged to clearly separate phases. The hexane supernatant containing chlorophyll was discarded. This process was repeated twice more. Absorbances at 535 nm with a baseline correction at 750 nm were measured using a Nanodrop 2000 Spectrophotometer (Thermo Scientific, Waltham, MA, USA).

For extraction of cannabinoids and cannflavins, freeze dried leaf disks or seedlings were weighed using a four‐place analytical balance (Mettler Toledo, Mississauga, ON, Canada), then homogenized at 30 s^−1^ for 1 min using a Retsch MM 400 Mixer Mill. The powder was then extracted (40 µL per milligram) with 9:1:0.001 methanol:dichloromethane:acetic acid and sonicated for 30 min at 40 kHz. Following sonication, extracts were centrifuged at 12,000 × g for 1 min. 1 part leaf disk extract was diluted in 1 part 9:1:0.001 methanol:dichloromethane:acetic acid and 4 parts 3:1:0.001 water:acetonitrile:acetic acid, and 1 part seedling extract was diluted in 2 parts 3:1:0.001 water:acetonitrile:acetic acid. Samples were vortexed briefly, incubated at room temperature for 5 min, spun down at 17,000 × g for 5 min, then spun through 0.22 µm PVDF at 12,000 × g for 2 min. Diluted extracts were sonicated for an additional 5 min prior to analysis by UPLC.

### 2.9 UPLC‐PDA

Cannabinoid and cannflavin metabolite measurements were performed using a Vanquish UPLC system (Thermo Scientific) equipped with a Quaternary Pump F, Split Sampler FT Autosampler, and Diode Array Detector HL (PDA). 1 µL injections were separated with an Acquity UPLC BEH shield RP18 column (2.1 mm i.d. × 150 mm, 1.7 µm particle size; Waters, Milford, MA, USA) with an Acquity UPLC BEH shield RP18 VanGuard precolumn (2.1 mm i.d. × 5 mm, 1.7 µm particle size; Waters) using a protocol modified from [19, 20]. The temperature of the column oven was 35°C. The solvents, at a flow rate of 300 µL/min, were eluent A (water with 0.1% acetic acid (*v*/*v*)) and eluent B (acetonitrile with 0.1% acetic acid (*v*/*v*)). The gradient: 0 to 3 min, 50% B; 3 to 8 min, 50% to 100% B; 8 to 18 min, 100% B, 18 to 20 min, 100% to 50% B, and 20 to 28 min, 50% B. The quantity of each metabolite was determined by comparison of the area under their peak at 263 nm absorbance in comparison to a standard curve of authentic standard integrated using the Chromeleon software (v7.2.10). The limit of detection was defined as the lowest amount of standard that produced a signal greater than three times the average blank signal at the corresponding retention time (Table S2). Recovery, injection precision, and inter‐day repeatability of the UPLC‐PDA method were evaluated using spiked leaf disk and seedling extracts. Detailed validation data are provided in Tables S3 and S4.

### 2.10 LC–MS

LC–MS/MS analysis was performed to confirm the identities of cannabinoids and cannflavins detected by UPLC‐PDA and was not intended for quantitative metabolite determination (Table S5). LC–MS/MS analysis was performed using a ZenoTOF 7600 mass spectrometer (SCIEX, Framingham, MA, USA) coupled to a UPLC M‐Class system (Waters, Milford, MA, USA). The precursor ions, qualifier ions, retention times, and mass accuracy used for compound identification are summarized in Table S5. Metabolites were separated on a Kinetex F5 column (2.6 µm, 100 Å, 150 × 0.3 mm; Phenomenex, Torrance, CA, USA). Samples were submitted in 50 µL of a solvent mixture consisting of one part 1:1:0.001 methanol:dichloromethane:acetic acid and two parts 3:1:0.001 water:acetonitrile:acetic acid, then diluted twofold with water containing 0.1% formic acid to reduce the organic solvent content to ≤50% prior to analysis. Mobile phase A consisted of water containing 0.1% formic acid, and mobile phase B consisted of acetonitrile containing 0.1% formic acid. Samples (3 µL) were separated at a flow rate of 8 µL min^−^^1^ using the following gradient: 1% B (0–0.5 min), 50%–60% B (0.5–10.5 min), 60%–100% B (10.5–11.0 min), 100% B (11.0–14.0 min), 100%–1% B (14.0–14.5 min), and 1% B (14.5–17.0 min), giving a total run time of 17 min. Mass spectra were acquired in positive ionization mode using MRM‐HR with an MS^1^ survey scan (*m*/*z* 100–1000) followed by targeted MS/MS acquisition. General ion source and acquisition parameters are summarized in Table S6, and compound‐specific MRM‐HR transitions are provided in Table S7. Because CBCA, CBDA, and THCA‐A, as well as CBDVA and THCVA, are isobaric compounds with identical molecular formulas, three MRM‐HR transitions were monitored for each group, and compound identities were confirmed using the corresponding UPLC‐UV chromatograms. All remaining analytes were identified using two MRM‐HR transitions.

### 2.11 Statistical Analyses

Statistical significance was determined using a two‐tailed *t*‐test assuming unequal variance in Excel v2512, or by a single factor ANOVA at an α value of 0.05 followed by Tukey's post hoc test. For qRT‐PCR, statistics were performed on biological replicates, which represented an average of up to four technical replicates.

## 3 Results

### 3.1 Transient Gene Expression in High‐THC *Cannabis*—the Leaf Disk Method

To determine whether genes can be transiently expressed in high‐THC strains of *Cannabis*, mature leaves of Megalodon × 50/50 Diesel (MD) and PC were infiltrated with *A. tumefaciens* EHA105 (*pSoup‐P19*) harboring the *RUBY* reporter cassette downstream of the viral *p35S* constitutive promoter. Red pigmentation, indicative of *RUBY* expression, was readily observed at infiltration sites in MD leaves at 6 days post infiltration (dpi; Figure 1A) but was absent from empty vector controls. Similar red pigmentation was observed in PC leaves, although the affected area surrounding each infiltration site was generally smaller than in MD (Figure S1).

**FIGURE 1:**
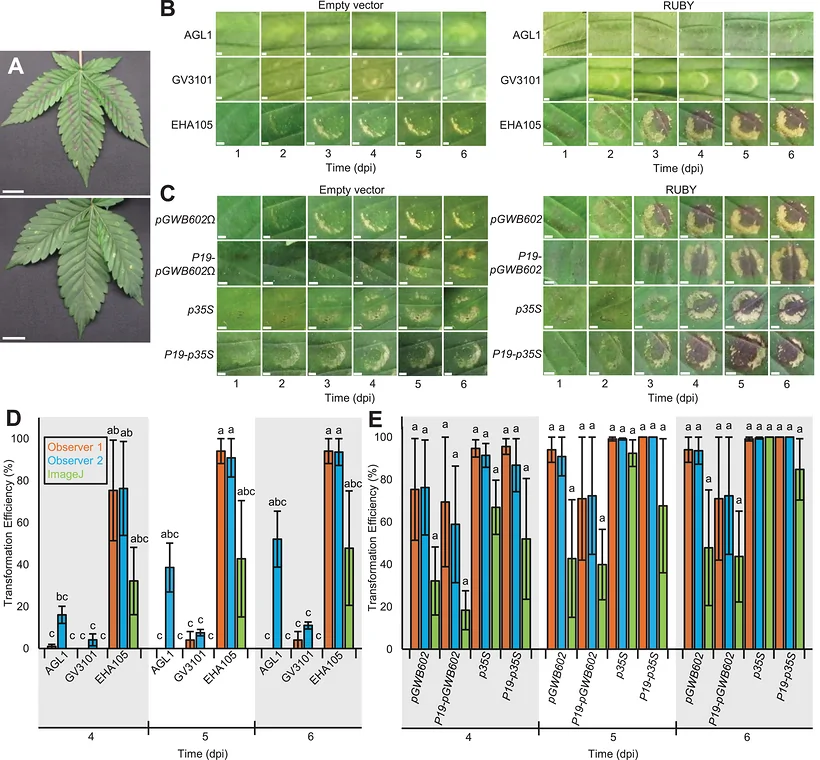
EHA105 and vector configuration independently influence transient transformation efficiency in mature leaves of high‐THC *Cannabis*. (A) Mature leaves of *Cannabis sativa* cultivar ‘MD’ infiltrated with EHA105 carrying *p35S::RUBY* (top) or the corresponding empty vector (bottom) at 6 days post‐infiltration (dpi). Scale bars = 2 cm. (B) Representative infiltration sites of mature ‘MD’ leaves infiltrated with AGL1, GV3101, or EHA105 carrying the common *pGWB602‐RUBY* construct or the corresponding empty vector control at 1–6 dpi. (C) Representative infiltration sites of mature ‘MD’ leaves infiltrated with EHA105 carrying *pGWB602‐RUBY*, *P19‐pGWB602‐RUBY*, *p35S::RUBY*, or *P19‐p35S::RUBY*, together with their corresponding empty vector controls, at 1–6 dpi. Scale bars in (B–C) = 1 mm. (D) Transformation efficiencies of AGL1, GV3101, and EHA105 expressing the common *pGWB602‐RUBY* construct at 4–6 dpi. (E) Transformation efficiencies of the four *RUBY* vector configurations expressed in the common EHA105 background at 4–6 dpi. Transformation efficiency was independently assessed by two observers and by ImageJ‐based image analysis. Values represent the mean ± SE (*n* = 3 biological replicates), where each biological replicate represents the transformation efficiency calculated from all infiltration sites across a single leaf (three leaflets). Statistical significance was determined by one‐way ANOVA followed by Tukey's post hoc test (*α* = 0.05). Different letters indicate significant differences (*p* < 0.05). Error bars are omitted where all biological replicates had identical values.

To determine whether the high transformation efficiency was attributable to the *Agrobacterium* strain itself, we compared AGL1, GV3101, and EHA105 expressing the same *pGWB602‐RUBY* construct. Visible red pigmentation developed most rapidly and extensively in leaves infiltrated with EHA105, whereas GV3101 and AGL1 produced substantially weaker pigmentation throughout the time course (Figure 1B).

Because the original EHA105 system differed from the other strains in both vector configuration and the presence of the RNA silencing suppressor P19, we next evaluated four plasmid combinations within the same EHA105 background. These included *pGWB602*, *P19‐pGWB602*, *p35S*, and *P19‐p35S* (Figure 1C). Red pigmentation was strongest in plants expressing *P19‐p35S*, followed by *p35S*, whereas addition of *P19* to the *pGWB602* construct produced little visible improvement.

Transformation efficiency was quantified by scoring the proportion of infiltration sites exhibiting red pigmentation at 4–6 dpi. Each leaf represented one biological replicate, with pigmentation scored independently by two observers and by ImageJ (see Methods). When the same *pGWB602‐RUBY* construct was compared among *Agrobacterium* strains, all three scoring methods consistently identified EHA105 as the most efficient strain at every time point (Figure 1D). Greater variability among the scoring methods was observed at 4 and 5 dpi, when betalain pigmentation was relatively weak and more difficult to distinguish from background tissue. By 6 dpi, pigmentation was readily distinguishable, and all three scoring methods converged, with EHA105 achieving 92%–94% transformation efficiency, whereas AGL1 and GV3101 remained below approximately 50%.

Using EHA105 as the host strain, transformation efficiencies of the vectors *p35S* or *pGWB602*, with and without *P19* were then compared (Figure 1E). By 5–6 dpi, all vector combinations produced up to 100% transformation efficiency. However, *p35S* and *P19‐p35S* showed the least variability, with nearly all leaves showing 100% transformation.

Collectively, these results demonstrate that both the EHA105 strain and vector contribute to the exceptionally high transformation efficiencies observed in leaf disks of high‐THC *Cannabis*.

### 3.2 *Agrobacterium* Strain and Plasmid Configuration Independently Influence Transgene Expression in High‐THC *Cannabis* Leaf Disks

To determine whether differences in transformation efficiency were accompanied by differences in transgene expression, *DODA*, one of the three genes comprising the *RUBY* reporter cassette (Figure 2A), was quantified by qRT‐PCR at 6 dpi. When the same *pGWB602‐RUBY* construct was expressed in different *Agrobacterium* strains, AGL1 produced significantly higher *DODA* transcript levels than either GV3101 or EHA105 (Figure 2B). However, betalain accumulation, assessed by pigment absorbance at 535 nm, was comparable between AGL1‐ and EHA105‐infiltrated leaf disks and significantly greater than that observed for GV3101 (Figure 2C,D).

**FIGURE 2:**
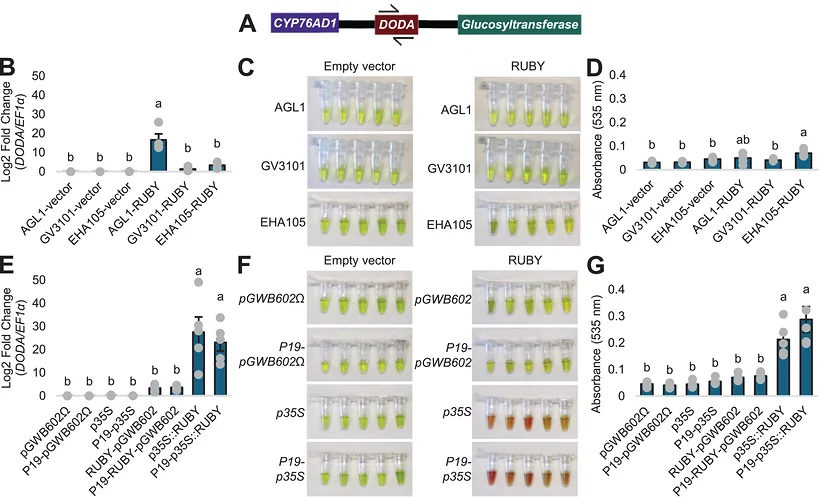
*Agrobacterium* strain and vector configuration independently influence transgene expression in mature leaves of high‐THC *Cannabis*. (A) Schematic of the *RUBY* reporter cassette showing the qRT‐PCR primer binding sites within the *DODA* coding sequence. Black lines indicate P2A self‐cleaving peptide sequences. Diagram created using BioRender. (B) Relative *DODA* transcript abundance, normalized to *Cannabis sativa TRANSLATION ELONGATION FACTOR 1 ALPHA 1* (*CsEF1α*), in leaf disks infiltrated with AGL1, GV3101, or EHA105 expressing the common *pGWB602‐RUBY* construct or the corresponding empty vector control at 6 dpi. Values represent the mean ± SE (*n* = 4 for *AGL1‐RUBY‐pGWB602*; *n* = 5 for all other treatments). (C) Representative metabolite extracts prepared from the corresponding leaf disks shown in (B) at 6 dpi. (D) Absorbance of the extracts shown in (C) measured at 535 nm. Values represent the mean ± SE (*n* = 5). (E) Relative *DODA* transcript abundance, normalized to *CsEF1α*, in leaf disks infiltrated with EHA105 expressing *pGWB602‐RUBY*, *P19‐pGWB602‐RUBY*, *p35S::RUBY*, or *P19‐p35S::RUBY*, together with their corresponding empty vector controls, at 6 dpi. Values represent the mean ± SE (*n* = 5). (F) Representative metabolite extracts prepared from the corresponding leaf disks shown in (E) at 6 dpi. (G) Absorbance of the extracts shown in (F) measured at 535 nm. Values represent the mean ± SE (*n* = 5). In (B), (D), (E), and (G), statistical significance was determined by one‐way ANOVA followed by Tukey's post hoc test (*α* = 0.05). Different letters indicate significant differences (*p* < 0.05).

To independently evaluate the contribution of plasmid configuration, the *Agrobacterium* background was held constant by expressing four *RUBY* vector configurations in EHA105. Specifically, *p35S* and *pGWB602* vectors were compared in the presence and absence of the RNA silencing suppressor *P19*. The *p35S::RUBY* construct produced the highest *DODA* transcript abundance, although transcript levels were not significantly different from those obtained with *P19‐p35S::RUBY* (Figure 2E). In contrast, *pGWB602‐RUBY* with or without *P19* resulted in substantially lower transcript accumulation. Betalain pigmentation of the corresponding leaf extracts closely paralleled the qRT‐PCR results, with *p35S::RUBY* and *P19‐p35S::RUBY* producing the strongest visible pigmentation and the highest absorbance at 535 nm (Figure 2F,G).

Collectively, these results demonstrate that both *Agrobacterium* strain and plasmid configuration influence transgene expression in high‐THC *Cannabis*. While AGL1 produced the highest *DODA* transcript levels when expressing the *pGWB602‐RUBY* construct, the *p35S*‐based vectors consistently outperformed the *pGWB602*‐based vectors within the common EHA105 background. Together with the transformation efficiency data presented in Figure 1, these findings indicate that the high performance of the EHA105 (*p35S*/*P19*) system reflects contributions from both the *Agrobacterium* host and the vector configuration.

### 3.3 Transient Gene Expression in High‐THC *Cannabis*—the Seedling Method

To expand the range of tissues amenable to gene function analysis in high‐THC *Cannabis*, we developed a transient gene expression method using whole seedlings. Given that EHA105 (*pSoup‐P19*)‐*p35S::RUBY* gave the best performance in the leaf disk method, we focused on this system to develop the seedling transformation method. Following dissection of embryos from germinated seeds (see Methods), we sonicated embryos with EHA105 (*pSoup‐P19*)‐*p35S::RUBY*. At 8 dpi, the leaf veins, hypocotyl and some of primary root of 81%–100% of *RUBY*‐infiltrated seedlings exhibited some degree of a red phenotype (Figure 3A). qRT‐PCR confirmed *DODA* overexpression in *RUBY‐*infiltrated seedlings (Figure 3B). Metabolite extracts of *RUBY*‐infiltrated seedlings were visually red in color and had significantly higher absorbance at 535 nm compared to the control (Figure 3C,D). Thus, EHA105 (*pSoup‐P19*) efficiently overexpresses transgenes in various organs of *Cannabis* seedlings.

**FIGURE 3:**
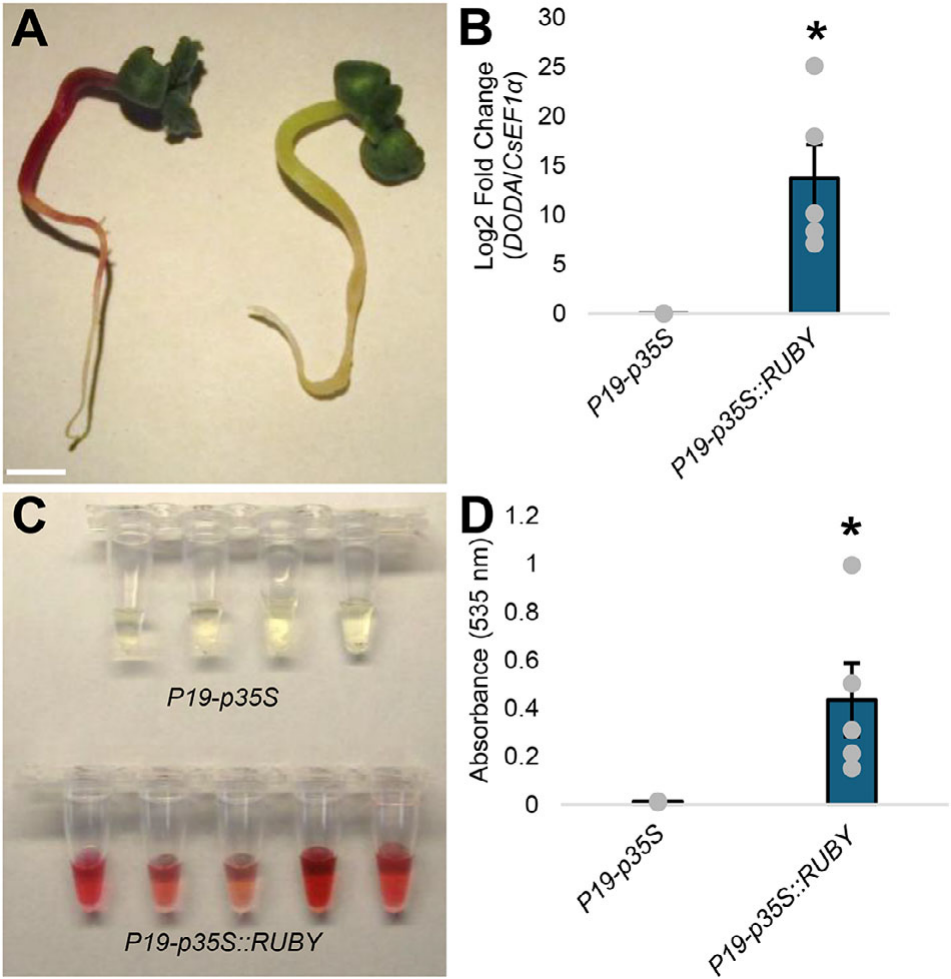
Efficient transient transgene expression in high‐THC *Cannabis* seedlings following agroinfiltration with EHA105 carrying *p35S::RUBY*. (A) Representative seedlings infiltrated with EHA105 carrying *p35S::RUBY* (left) or the corresponding empty vector control (right) at 8 days post infiltration (dpi). Scale bar = 0.5 cm. (B) Relative *DODA* transcript abundance, normalized to *Cannabis sativa TRANSLATION ELONGATION FACTOR 1 ALPHA 1* (*CsEF1α*), determined by qRT‐PCR. Values represent the mean ± SE (*n* = 4 for the empty vector control and *n* = 5 for *p35S::RUBY*). (C) Representative metabolite extracts prepared from seedlings shown in (A). (D) Absorbance of the extracts shown in (C) measured at 535 nm. Values represent the mean ± SE (*n* = 4 for the empty vector control and *n* = 5 for *p35S::RUBY*). In (B) and (D), statistical significance was determined using a two‐tailed Welch's t‐test (unequal variances). *p* ≤ 0.05.

### 3.4 Measuring Cannabinoid and Cannflavin Metabolites From Leaf Disk and Seedling Extracts by UPLC‐PDA

Given the growing interest in understanding the genetics underlying specialized metabolism in *Cannabis*, particularly in trichome‐bearing tissues, we assessed whether leaf disks and seedlings contain trichomes. Microscopy demonstrated that MD leaf disks contain peltate glandular trichomes (Figure 4A). By contrast, seedling hypocotyls had hair‐like, nonglandular trichomes (Figure 4B).

**FIGURE 4:**
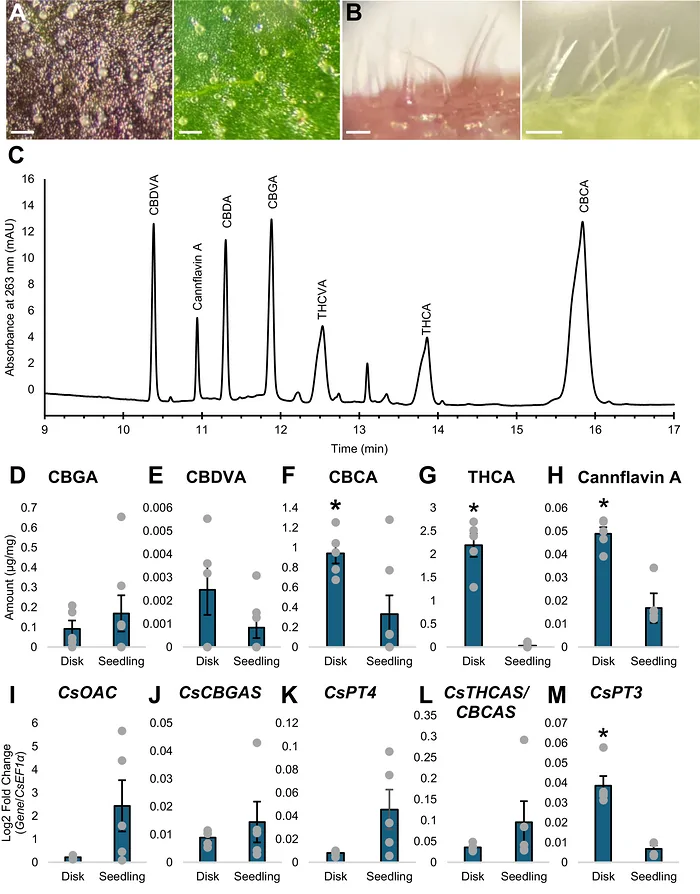
Comparison of leaf disk and seedling agroinfiltration systems in high‐THC *Cannabis*. (A) Representative glandular peltate trichomes on mature leaves infiltrated with EHA105 carrying *p35S::RUBY* (left) or the corresponding empty vector control (right). (B) Representative nonglandular trichomes on hypocotyls of seedlings infiltrated with EHA105 carrying *p35S::RUBY* (left) or the corresponding empty vector control (right). Scale bars = 100 µm. (C) Representative UPLC‐PDA chromatogram of a seedling extract spiked with a 20 µM mixed standard. Peaks corresponding to CBGA, CBDVA, CBCA, THCA, and cannflavin A are labelled. (D–H) Quantification of CBGA, CBDVA, CBCA, THCA, and cannflavin A in leaf disks and seedlings infiltrated with the EHA105 empty vector control. Values represent the mean ± SE (*n* = 5 for leaf disks and *n* = 7 for seedlings). (I–M) Relative transcript abundance of cannabinoid biosynthetic genes *CsOAC*, *CsCBGAS*, *CsPT4*, *CsTHCAS*/*CBCAS*, and the cannflavin biosynthetic gene *CsPT3*, normalized to *Cannabis sativa TRANSLATION ELONGATION FACTOR 1 ALPHA 1* (*CsEF1α*), in leaf disks and seedlings infiltrated with the EHA105 empty vector control. Values represent the mean ± SE (*n* = 4 for seedling *CsPT3* and *n* = 5 for all other treatments). In (D–M), statistical significance was determined using a two‐tailed Welch's t‐test (unequal variances). Asterisks indicate significant differences (*p* < 0.05).

In attempt to measure cannabinoid and cannflavin metabolites, we developed a UPLC‐PDA protocol by spiking cannabinoid and cannflavin authentic standards into seedling extracts (see Methods). After several rounds of optimization, the protocol readily measured the cannabinoids CBDVA, CBDA, CBGA, THCVA, THCA, CBCA, and flavonoid cannflavin A (Figure 4C). The identities of these metabolites were confirmed independently by LC–MS/MS based on accurate precursor masses, qualifier ions, and retention times (Table S5). The limit of detection by UPLC‐PDA [21] was 0.03125, 0.5, 0.25, 0.5, 0.5, 2, and 0.0625 µM, respectively, and the response was linear up to the highest concentration measured (50 µM). This LOD equates to 8.95 × 10^−6^, 1.79 × 10^−4^, 9.01 × 10^−5^, 1.43 × 10^−4^, 1.79 × 10^−4^, 7.17 × 10^−4^, and 2.92 × 10^−5^ µg, respectively (see Methods).

Upon testing acetone:acetic acid (1:0.001), acetonitrile:acetic acid (1:0.001), and methanol:dichloromethane:acetic acid (9:1:0.001), the optimal extraction solvent for detecting cannabinoid and cannflavin while minimizing background was methanol:dichloromethane:acetic acid (9:1:0.001). The optimal dilution to maximize signal‐to‐noise ratio was six‐ and threefold for leaf disk and seedling extracts, respectively. It may be noteworthy that we injected at least one blank between each sample and washed the column with acetonitrile after each experiment to minimize buildup in the column.

With the optimized protocol, we measured four cannabinoids and cannflavin A from MD leaf disk and seedling extracts. Leaf disks contained primarily THCA and CBCA (2.19 and 0.94 µg mg^−1^ tissue), whereas seedlings had primarily CBGA and CBCA (0.17 and 0.33 µg mg^−1^) (Figure 4D–H). Notably, leaf disks had significantly greater amounts of CBCA, THCA, and cannflavin A.

The expression of several cannabinoid and cannflavin biosynthetic genes was detectable from leaf disks and seedlings by qRT‐PCR (Figure 4I,M). The four cannabinoid biosynthetic genes did not exhibit significantly different expression between leaf disk and seedlings (Figure 4I–L). This is consistent with CBGA and CBDVA metabolite amounts being similar between the two tissues (Figure 4D–G). While THCA amounts were elevated in leaf disks, *CsTHCAS/CBCAS* expression was not, similar to a previous report on dried flower materials [22] and fresh flowers of the high‐THC strain “Gorilla Glue” [23]. Significantly higher expression of cannflavin biosynthetic gene *CsPT3* was observed in leaf disks (Figure 4M), consistent with greater amounts of cannflavin metabolite (Figure 4H).

Overall, our data demonstrate easy, robust protocols for measuring the expression of cannabinoid and cannflavin A metabolites and genes, paving the way for gene function studies in *Cannabis*.

## 4 Discussion

The lack of robust genetic tools has remained a major limitation for functional genomics and metabolic engineering in *Cannabis*. In this study, we have developed two *Agrobacterium*‐mediated agroinfiltration protocols that enable rapid and reproducible transient gene expression in trichome‐bearing *Cannabis* tissues. Together, these approaches address a persistent technical bottleneck in *Cannabis* biotechnology by providing an accessible alternative to stable transformation systems.

### 4.1 Transient Gene Expression in *Cannabis*

*Cannabis* is widely regarded as recalcitrant to genetic transformation, with previous transient gene expression studies reporting low or inconsistent efficiencies, strong genotype dependence, or limited applicability across tissue types [6, 24, 25]. In the present study, we developed two *Agrobacterium*‐mediated transient expression protocols that enable rapid, reproducible transgene expression in commercially relevant, high‐THC *Cannabis* cultivars. Compared with previously published *Agrobacterium*‐based transient transformation systems, our leaf disk protocol achieved transformation efficiencies of up to 100%, while the seedling protocol reached 81%–100% transformation efficiency. Together with the comparison presented in Table 1, these findings establish one of the highest‐efficiency *Agrobacterium*‐mediated transient expression platforms currently reported for drug‐type *Cannabis*.

Unlike previous studies that primarily compared complete transformation systems (Table 1), we independently evaluated the contributions of *Agrobacterium* strain and plasmid configuration. Holding the binary vector constant demonstrated that transformation efficiency differed substantially among *Agrobacterium* strains, with EHA105 consistently producing the highest proportion of transformed infiltration sites. Conversely, when *Agrobacterium* background was held constant, plasmid configuration strongly influenced reporter gene expression, with the *p35S*‐based constructs producing substantially greater *RUBY* expression than the *pGWB602*‐based vectors. These experiments demonstrate that transformation efficiency and transgene expression are influenced by distinct experimental variables and should therefore be evaluated independently.

Although AGL1 produced the highest *DODA* transcript abundance when expressing the *pGWB602‐RUBY* construct, *EHA105* consistently produced the highest transformation efficiency and comparable betalain accumulation. These findings indicate that transformation efficiency and transgene expression represent distinct characteristics of transient expression systems and should therefore be evaluated independently.

The present study was designed to establish a robust and reproducible transient expression workflow rather than to systematically optimize individual infiltration parameters. Accordingly, variables such as bacterial density, acetosyringone concentration, post‐infiltration dark incubation, and leaf developmental stage were held constant throughout the experiments. Further optimization of these parameters may improve performance for other *Cannabis* genotypes or experimental applications.

### 4.2 Enhanced Transgene Expression Capacity

Beyond transformation efficiency, the magnitude of transgene expression is a key determinant of the utility of transient expression systems for functional genomics. Our results demonstrate that both *Agrobacterium* strain and vector configuration influence transgene expression. When expressing the common *pGWB602‐RUBY* construct, AGL1 produced the highest *DODA* transcript levels, while both AGL1 and EHA105 accumulated similarly high levels of betalain pigment, exceeding those observed with GV3101. AGL1 and EHA105 are both supervirulent strains derived from the agropine‐type pTiBo542 tumor‐inducing (Ti) plasmid and are widely used for transformation of recalcitrant plant species, whereas GV3101 is derived from the nopaline‐type pTiC58 Ti plasmid and is generally preferred for rapid, high‐efficiency transformation of *Arabidopsis thaliana* [26, 27].

Holding the *Agrobacterium* background constant revealed that vector configuration exerted an even greater influence on transgene expression. Within EHA105, the *p35S*‐based vectors consistently produced substantially higher *RUBY* transcript levels and betalain accumulation than the *pGWB602*‐based vectors. Together with the superior transformation efficiency of EHA105, these findings indicate that the exceptional performance of the EHA105 (*pSoup‐P19*) system reflects contributions from both the *Agrobacterium* host and vector design. Although the *p35S* and *pGWB602* vectors are similar in overall size, contain comparable T‐DNA regions, and both utilize the *CaMV 35S* promoter to drive transgene expression [13, 28], they differ substantially in overall vector architecture. Taken together, our results suggest that differences in vector architecture, rather than promoter identity or plasmid size alone, contribute to the observed differences in transgene expression.

Interestingly, although *p35S::RUBY* produced the highest *DODA* transcript abundance, transcript levels were not significantly different from those obtained with *P19‐p35S::RUBY* (Figure 2E). In contrast, *P19‐p35S::RUBY* consistently accumulated slightly higher levels of betalain pigment (Figure 2G). While this difference was modest, it suggests that P19 may enhance reporter output through post‐transcriptional mechanisms in addition to its effects on transcript accumulation, consistent with its established role as a suppressor of RNA silencing that reduces degradation of transgene‐derived RNAs [29, 30].

Overall, our findings demonstrate that efficient transient expression in *Cannabis* depends on both successful T‐DNA delivery and robust transgene expression. Although the molecular basis underlying the superior performance of the EHA105 (*pSoup‐P19*) system remains to be determined, the combination of high transformation efficiency and strong reporter expression establishes it as a highly effective platform for *Cannabis* functional genomics.

### 4.3 Complementary Transient Expression Platforms

A major advantage of the methods presented here is the availability of two complementary transient expression platforms. Mature leaf disks provide an experimentally convenient system containing glandular trichomes, the specialized structures responsible for cannabinoid biosynthesis. Consequently, leaf disks are particularly well suited for investigating genes involved in cannabinoid and cannflavin metabolism.

By contrast, the seedling protocol enables rapid gene expression studies within days of germination and avoids the time and resources required to cultivate mature plants. Although seedlings contain primarily nonglandular trichomes and lower cannabinoid concentrations, they provide a rapid platform for evaluating gene expression constructs, promoters, genome‐editing reagents, and regulatory proteins prior to more detailed analyses in mature tissues. Together, these complementary systems provide considerable experimental flexibility depending on the biological question being addressed.

Interestingly, *CsTHCAS*/*CBCAS* transcript abundance did not directly correlate with THCA metabolite accumulation between leaf disks and seedlings. This lack of correlation is not unexpected, as THCA biosynthesis is regulated at multiple levels beyond transcription. Previous studies have demonstrated that THCAS expression is highly restricted to the secretory disc cells of glandular trichomes, where the enzyme undergoes post‐translational processing, including N‐linked glycosylation, before being secreted into the extracellular storage cavity in which the final conversion of CBGA to THCA occurs [31, 32, 33]. Consequently, THCA accumulation is influenced not only by transcript abundance, but also by trichome abundance, enzyme maturation, secretion, and compartmentalization within glandular trichomes. These factors likely contribute to the differences in THCA accumulation observed between mature leaf disks and seedlings, which differ substantially in developmental stage and trichome composition.

### 4.4 Relevance for Specialized Metabolism Studies

Functional analysis of specialized metabolic pathways in *Cannabis* presents unique challenges due to tissue‐ and cell type‐specific metabolite accumulation. Cannabinoid biosynthesis is largely restricted to glandular trichomes, limiting the utility of alternative systems such as hairy root cultures [8]. The demonstration that leaf disks contain glandular trichomes, express cannabinoid biosynthetic genes, and accumulate cannabinoid metabolites confirms that agroinfiltrated leaf tissues provide a biologically relevant platform for interrogating the genetics of cannabinoid production.

Furthermore, the detection of cannflavins in both leaf and seedling tissues highlights the broader applicability of these systems. While metabolite levels differ across developmental stages, the presence of measurable compounds suggests that transient manipulation of gene expression can be integrated with biochemical measurements to study specialized metabolism in *Cannabis*. This capability is particularly valuable for studies of gene regulation, gene function, and metabolic flux.

The biochemical differences observed between leaf disks and seedlings should also be interpreted in the context of their distinct developmental states. Mature leaves contain abundant glandular peltate trichomes, the primary sites of cannabinoid and cannflavin biosynthesis, whereas young seedlings possess predominantly nonglandular trichomes and are at an earlier developmental stage. Consequently, differences in metabolite abundance and biosynthetic gene expression between these tissues were expected, and agree with previous reports of higher cannabinoid accumulation in mature leaf tissues compared to seedlings [34], and should not be interpreted as differences in transformation performance. Rather, our comparisons highlight the complementary applications of the two systems: leaf disks provide a physiologically relevant platform for studies of specialized metabolism, whereas seedlings offer a rapid and experimentally convenient system for evaluating gene function and transgene expression prior to analyses in mature tissues.

### 4.5 An Integrated Platform for Functional Genomics

One objective of this study was not simply to develop a transient transformation protocol, but to establish an integrated workflow linking gene manipulation with metabolite analysis. To complement the transformation methods, we developed a single UPLC‐PDA protocol capable of simultaneously measuring multiple cannabinoids and cannflavins within the same chromatographic run. LC–MS/MS analysis confirmed the identities of the detected metabolites, whereas quantitative measurements were performed using the UPLC‐PDA platform.

Analytical workflows for *Cannabis* metabolites frequently rely on LC–MS or GC‐MS instrumentation, which provide superior sensitivity and structural information but require specialized instrumentation, greater operational costs, and more complex data analysis. The UPLC‐PDA method described here was not intended to replace mass spectrometry‐based analyses. Rather, it provides an accessible alternative for studies requiring comparative metabolite measurements, such as transient expression assays, functional characterization of biosynthetic genes, and metabolic engineering experiments. By enabling simultaneous quantification of both cannabinoids and cannflavins using standard chromatographic instrumentation, the method substantially simplifies experimental workflows.

### 4.6 Applications for *Cannabis* Biotechnology

Reliable transient expression systems have broad utility throughout *Cannabis* biotechnology. They provide a rapid means to evaluate candidate genes, characterize transcription factors, assess promoter activity, optimize genome‐editing reagents, and investigate specialized metabolic pathways before committing to considerably more challenging stable transformation experiments. Because these assays require only days rather than months, they substantially accelerate hypothesis testing.

Importantly, the combination of high‐efficiency transient transformation with a streamlined metabolite analysis pipeline enables molecular and biochemical phenotypes to be evaluated within the same experimental framework. This integrated approach should facilitate studies of cannabinoid biosynthesis, metabolic regulation, and synthetic biology while remaining accessible to laboratories lacking specialized transformation or mass spectrometry infrastructure.

## 5 Conclusion

This study establishes an integrated experimental framework that expands the molecular and analytical toolkit available for *Cannabis*. We developed two *Agrobacterium*‐mediated agroinfiltration protocols that enable rapid, robust, and reproducible transient gene expression in *Cannabis* leaf and seedling tissues. These methods provide an accessible alternative to stable transformation systems and overcome longstanding barriers associated with genotype dependence and transformation recalcitrance.

In parallel, we introduce a single UPLC‐PDA method capable of simultaneously measuring cannabinoids and cannflavins. This analytical platform offers a simple, cost‐effective approach for metabolite quantification that is readily adaptable to functional genomics and metabolic engineering workflows.

Together, these complementary advances establish a practical dual‐platform system that links efficient transient gene manipulation with streamlined biochemical analysis. This integrated capability enables rapid interrogation of gene function, regulatory mechanisms, and specialized metabolic pathways, thereby facilitating scalable molecular studies in *Cannabis*. The approaches described here provide a foundation for accelerated gene discovery, pathway characterization, and biotechnology‐driven trait optimization in this increasingly important medicinal crop.

## Author Contributions

**Nik Kovinich**: conceptualization, methodology, supervision, funding acquisition, project administration, writing – original draft, writing – review and editing. **Sarah Pullano**: methodology, investigation, formal analysis, writing – original draft, writing – review and editing. **Aida Shahabi**: methodology, investigation, formal analysis, writing – original draft, writing – review and editing. **Parmis Shariat Torbaghani**: investigation, formal analysis, writing – review and editing. **Ivan Monsalvo**: methodology, investigation, formal analysis, writing – review and editing. **Madison Sandler**: investigation, formal analysis, writing – review and editing. **Adam Sumner**: writing – review and editing. **Alex Harris**: writing – review and editing. **David Haak**: funding acquisition, writing – review and editing. **Bastiaan Bargmann**: funding acquisition, writing – review and editing.

## Conflicts of Interest

The authors declare no conflicts of interest.

## Accession Numbers

*35S‐RUBY* (Addgene 160908).

## Supporting information




**Supporting File 1**: biot70316‐sup‐0001‐FigureS1.pptx.

**Supporting File 2**: biot70316‐sup‐0002‐Caption.docx.

**Supporting File 3**: biot70316‐sup‐0003‐TableS1.xlsx.

**Supporting File 4**: biot70316‐sup‐0004‐TableS2.xlsx.

**Supporting File 5**: biot70316‐sup‐0005‐TableS3.xlsx.

**Supporting File 6**: biot70316‐sup‐0006‐TableS4.xlsx.

**Supporting File 7**: biot70316‐sup‐0007‐TableS5.xlsx.

**Supporting File 8**: biot70316‐sup‐0008‐TableS6.xlsx.

**Supporting File 9**: biot70316‐sup‐0009‐TableS7.xlsx.

## Data Availability

The data that support the findings of this study are available from the corresponding author upon reasonable request.

## References

[1] S. Schilling , R. Melzer , and P. F. McCabe , “Cannabis sativa,” Current Biology 30 (2020): R8–R9, 10.1016/j.cub.2019.10.039. 31910378

[2] Z. Xie , Y. Mi , L. Kong , et al., “ *Cannabis sativa*: Origin and History, Glandular Trichome Development, and Cannabinoid Biosynthesis,” Horticulture Research 10 (2023): uhad150, 10.1093/hr/uhad150. PMC10485653 37691962

[3] S. Shujat , G. I. Robinson , F. Norouzkhani , and I. Kovalchuk , “Using Advanced Biotechnological Techniques to Improve *Cannabis* Cultivars,” Biocatalysis and Agricultural Biotechnology 60 (2024): 103250, 10.1016/j.bcab.2024.103250.

[4] Z. K. Punja , “Hop Latent Viroid‐Infected *Cannabis* (*Cannabis sativa* L.) Plants Show Enhanced Susceptibility to Fusarium Root and Stem Rot and Powdery Mildew Infection,” Canadian Journal of Plant Pathology 48 (2026): 1–15.

[5] S. M. Ahsan , M. D. Injamum‐Ul‐Hoque , N. C. Howlader , et al., “Haploid Production in *Cannabis sativa*: Recent Updates, Prospects, and Perspectives,” Biology 14 (2025): 701, 10.3390/biology14060701. PMC12189703 40563952

[6] M. Li , Q. Wu , F. Guo , et al., “A Versatile, Rapid *Agrobacterium*‐Mediated Transient Expression System for Functional Genomics Studies in *Cannabis* Seedling,” Planta 260 (2024): 18, 10.1007/s00425-024-04448-5. 38837044

[7] X. Zhang , G. Xu , C. Cheng , et al., “Establishment of an *Agrobacterium*‐Mediated Genetic Transformation and CRISPR/Cas9‐Mediated Targeted Mutagenesis in Hemp (*Cannabis sativa* L.),” Plant Biotechnology Journal 19 (2021): 1979–1987, 10.1111/pbi.13611. PMC8486249 33960612

[8] L. Matchett‐Oates , E. Mohamaden , G. Spangenberg , and N. Cogan , “Development of a Robust Transient Expression Screening System in Protoplasts of *Cannabis* ,” In Vitro Cellular & Developmental Biology‐Plant 57 (2021): 1040–1050.

[9] L. Ajdanian , M. Niazian , and D. Torkamaneh , “Optimizing Ex Vitro One‐Step RUBY‐Equipped Hairy Root Transformation in Drug‐ and Hemp‐Type *Cannabis* ,” Plant Biotechnology Journal 22 (2024): 1957–1959, 10.1111/pbi.14314. PMC11182590 38400583

[10] Q. Wang , D. Zhang , Y. R. Dai , and C. C. Liu , “Efficient Tobacco Rattle Virus‐Induced Gene Editing in Tomato Mediated by the CRISPR/Cas9 System,” Biotechnology Journal 19 (2024): 2400204, 10.1002/biot.202400204. 38797722

[11] S. Devi , A. Rani , N. Devi , et al., “Engineering an Agro‐Infectious Apple Stem Grooving Virus Clone for Virus‐Induced Gene Silencing (VIGS) in Plants,” Biotechnology Journal 20 (2025): 70063, 10.1002/biot.70063. 40544490

[12] P. Yuan , M. Usman , W. Liu , et al., “Advancements in Plant Gene Editing Technology: From Construct Design to Enhanced Transformation Efficiency,” Biotechnology Journal 19 (2024): 202400457, 10.1002/biot.202400457. PMC11653234 39692063

[13] Y. He , T. Zhang , H. Sun , H. Zhan , and Y. Zhao , “A Reporter for Noninvasively Monitoring Gene Expression and Plant Transformation,” Horticulture Research 7 (2020): 152, 10.1038/s41438-020-00390-1. PMC7502077 33024566

[14] B. Laflamme , M. Middleton , T. Lo , D. Desveaux , and D. S. Guttman , “Image‐Based Quantification of Plant Immunity and Disease,” Molecular Plant‐Microbe Interactions® 29 (2016): 919–924, 10.1094/MPMI-07-16-0129-TA. 27996374

[15] R. Sullivan , G. C. Paré , L. J. Frederiksen , G. L. Semenza , and C. H. Graham , “Hypoxia‐Induced Resistance to Anticancer Drugs Is Associated With Decreased Senescence and Requires Hypoxia‐Inducible Factor‐1 Activity,” Molecular Cancer Therapeutics 7 (2008): 1961–1973, 10.1158/1535-7163.MCT-08-0198. 18645006

[16] M. Deguchi , S. Potlakayala , Z. Spuhler , et al., “Selection and Validation of Reference Genes for Normalization of qRT‐PCR Data to Study the Cannabinoid Pathway Genes in Industrial Hemp,” PLoS ONE 16 (2021): 0260660, 10.1371/journal.pone.0260660. PMC8687539 34928958

[17] R. Guo , H. Guo , Q. Zhang , et al., “Evaluation of Reference Genes for RT‐qPCR Analysis in Wild and Cultivated *Cannabis* ,” Bioscience, Biotechnology, and Biochemistry 82 (2018): 1902–1910, 10.1080/09168451.2018.1506253. 30130459

[18] S. Ahmed , X. Gao , M. A. Jahan , M. Adams , N. Wu , and N. Kovinich , “Nanoparticle‐Based Genetic Transformation of *Cannabis sativa* ,” Journal of Biotechnology 326 (2021): 48–51, 10.1016/j.jbiotec.2020.12.014. 33373624

[19] K. Farrell , M. A. Jahan , and N. Kovinich , “Distinct Mechanisms of Biotic and Chemical Elicitors Enable Additive Elicitation of the Anticancer Phytoalexin Glyceollin I,” Molecules 22 (2017): 1261, 10.3390/molecules22081261. PMC6152012 28749423

[20] J. Lin , I. Monsalvo , M. Ly , et al., “RNA‐Seq Dissects Incomplete Activation of Phytoalexin Biosynthesis by the Soybean Transcription Factors GmMYB29A2 and GmNAC42‐1,” Plants 12 (2023): 545, 10.3390/plants12030545. PMC9921300 36771629

[21] X. Tang , M. Demiray , T. Wirth , and R. K. Allemann , “Concise Synthesis of Artemisinin From a Farnesyl Diphosphate Analogue,” Bioorganic & Medicinal Chemistry 26 (2018): 1314–1319, 10.1016/j.bmc.2017.03.068. PMC5930831 28404524

[22] F. Cascini , S. Passerotti , and S. Martello , “A Real‐Time PCR Assay for the Relative Quantification of the Tetrahydrocannabinolic Acid (THCA) Synthase Gene in Herbal *Cannabis* Samples,” Forensic Science International 217 (2012): 134–138, 10.1016/j.forsciint.2011.10.041. 22093702

[23] P. V. Apicella , L. B. Sands , Y. Ma , and G. A. Berkowitz , “Delineating Genetic Regulation of Cannabinoid Biosynthesis During Female Flower Development in *Cannabis sativa* ,” Plant Direct 6 (2022): 412, 10.1002/pld3.412. PMC9219008 35774623

[24] M. Deguchi , D. Bogush , H. Weeden , et al., “Establishment and Optimization of a Hemp (*Cannabis sativa* L.) Agroinfiltration System for Gene Expression and Silencing Studies,” Scientific Reports 10 (2020): 3504, 10.1038/s41598-020-60323-9. PMC7044430 32103049

[25] A. Sorokin , N. S. Yadav , D. Gaudet , and I. Kovalchuk , “Transient Expression of the β‐Glucuronidase Gene in *Cannabis sativa* Varieties,” Plant Signaling & Behavior 15 (2020): 1780037, 10.1080/15592324.2020.1780037. PMC8570765 32552427

[26] E. Aliu , Q. Ji , A. Wlazlo , et al., “Enhancing *Agrobacterium*‐Mediated Plant Transformation Efficiency through Improved Ternary Vector Systems and Auxotrophic Strains,” Frontiers in Plant Science 15 (2024): 1429353, 10.3389/fpls.2024.1429353. PMC11300283 39109064

[27] V. J. Chetty , N. Ceballos , D. Garcia , J. Narváez‐Vásquez , W. Lopez , and M. L. Orozco‐Cárdenas , “Evaluation of Four *Agrobacterium tumefaciens* Strains for the Genetic Transformation of Tomato (*Solanum lycopersicum* L.) Cultivar Micro‐Tom,” Plant Cell Reports 32 (2013): 239–247, 10.1007/s00299-012-1358-1. 23099543

[28] S. Nakamura , S. Mano , Y. Tanaka , et al., “Gateway Binary Vectors With the Bialaphos Resistance Gene, Bar , as a Selection Marker for Plant Transformation,” Bioscience, Biotechnology, and Biochemistry 74 (2010): 1315–1319, 10.1271/bbb.100184. 20530878

[29] S. Qian , X. Zhong , L. Yu , B. Ding , P. de Haan , and K. Boris‐Lawrie , “HIV‐1 Tat RNA Silencing Suppressor Activity Is Conserved Across Kingdoms and Counteracts Translational Repression of HIV‐1,” Proceedings of the National Academy of Sciences 106 (2009): 605–610, 10.1073/pnas.0806822106. PMC2626750 19122141

[30] D. C. Danielson and J. P. Pezacki , “Studying the RNA Silencing Pathway With the p19 Protein,” FEBS letters 587 (2013): 1198–1205, 10.1016/j.febslet.2013.01.036. 23376479

[31] M. Geissler , J. Volk , F. Stehle , O. Kayser , and H. Warzecha , “Subcellular Localization Defines Modification and Production of Δ9‐Tetrahydrocannabinolic Acid Synthase in Transiently Transformed *Nicotiana benthamiana* ,” Biotechnology Letters 40 (2018): 981–987, 10.1007/s10529-018-2545-0. 29619743

[32] F. Taura , E. Dono , S. Sirikantaramas , K. Yoshimura , Y. Shoyama , and S. Morimoto , “Production of Δ1‐Tetrahydrocannabinolic Acid by the Biosynthetic Enzyme Secreted from Transgenic *Pichia pastoris* ,” Biochemical and Biophysical Research Communications 361 (2007): 675–680, 10.1016/j.bbrc.2007.07.079. 17669365

[33] P. M. Siazon , M. Nolan , Q. Guo , R. Perovich , T. Kretzschmar , and J. C. Mieog , “Heads Up: Transcriptomics Reveal Functional Roles of *Cannabis* Glandular Trichome Stalks,” Plants 15 (2026): 1624, 10.3390/plants15111624. PMC13259226 42280661

[34] A. F. Vogelmann , J. C. Turner , and P. G. Mahlberg , “Cannabinoid Composition in Seedlings Compared to Adult Plants of *Cannabis sativa* ,” Journal of Natural Products 51 (1988): 1075–1079, 10.1021/np50060a004.

