# Supplementary material for: An Integrated Transient Expression and UPLC‐PDA Approach for Functional Genomics and Metabolic Analysis of Cannabis sativa
Source: Biotechnol J. 2026 Oct 14;21(10):e70316. doi: 10.1002/biot.70316 (PMC13669522; doi:10.1002/biot.70316)
Supplement: Supplementary file 1 [file BIOT-21-e70316-s008.docx]

**Supplementary Figure S1:** Strain-specific variation in leaflet agroinfiltration. (A) ‘PC’ and (B) **‘**MD’ centre leaflets infiltrated with EHA105 (*pSoup-P19*)-*p35S::RUBY*, 6 dpi.
